# A TabPFN-based prediction system for refractive error and dry eye comorbidity: a retrospective study using large-scale real-world data

**DOI:** 10.3389/fcell.2026.1770427

**Published:** 2026-04-09

**Authors:** Danyi Qin, Wenying Guan, Shinan Wu, Changsheng Xu, Yuwen Liu, Bing Yan, Jingyao Lv, Xiaoxin Li, Zuguo Liu

**Affiliations:** 1 Xiamen University affiliated Xiamen Eye Center, Fujian Provincial Key Laboratory of Ophthalmology and Visual Science, Fujian Engineering and Research Center of Eye Regenerative Medicine, School of Medicine, Eye Institute of Xiamen University, Xiamen University, Xiamen, Fujian, China; 2 Department of Ophthalmology, Hengyang Medical School, The First Affiliated Hospital of University of South China, University of South China, Hengyang, Hunan, China

**Keywords:** artificial intelligence, clinical decision system, dry eye, machine learning, refractive error

## Abstract

**Introduction:**

Refractive error and dry eye are highly prevalent ocular conditions that significantly impair the quality of life and impose a substantial burden on individuals and society. Growing evidence suggests a correlation between these two conditions. This study aimed to develop and validate a machine learning (ML) model to accurately predict the risk of concurrent dry eye comorbidities in patients with refractive error.

**Methods:**

Data from Xiamen Eye Center outpatient database (1st January 2024 to 28th February 2025) were analyzed (n = 114,579). Hyperparameter optimization, *Spearman* correlation analysis, and logistic regression analyses were performed. The final feature set was determined using a Random Forest algorithm with the sequential forward selection technique. Eight ML algorithms were evaluated through ten-fold cross-validation. The optimal model was selected based on a comprehensive assessment of the receiver operating characteristic curve, precision-recall curve, and decision curve analysis. For the best-performing model, SHapley Additive exPlanations and partial dependence plots were utilized to interpret the importance and interactions of risk factors.

**Results:**

Baseline characteristics were comparable between the training set and the internal test set, while significant differences were observed in multiple baseline characteristics between dry eye group and non-dry eye group among subjects with refractive error. Based on ten selected feature variables, the tabular prior-data fitted network (TabPFN) model demonstrated the best performance, showing high screening efficacy with both specificity and accuracy reaching 0.945. The interaction analysis revealed that a longer duration of refractive error was associated with a higher risk of dry eye, a relationship that was particularly pronounced among older and female patients. Furthermore, an online web calculator was developed to deploy this diagnostic prediction model.

**Discussion:**

This study developed a high-performance and interpretable ML system based on a large-scale real-world clinical dataset for the early prediction of concurrent dry eye risk in patients with refractive error. The system holds significant potential as a predictive aid for clinical decision-making, enabling more timely and personalized patient management, thereby offering substantial clinical value and promising application prospects.

## 1 Introduction

The etiology of refractive error (RE) is complex and multifactorial, involving genetic predisposition, poor visual habits (Hao et al., 2024), inflammatory and immune responses (Zhang et al., 2025), and ocular diseases (Wei et al., 2018; Wei et al., 2025). Uncorrected RE is the leading cause of visual impairment in children worldwide (Little et al., 2025). Myopia has reached a global prevalence affecting approximately one-third of children and adolescents (Liang et al., 2025), with increasing severity also observed in adults (Sankaridurg et al., 2021). It is projected that by 2050, the global myopic population will reach 4.758 billion, accounting for about 49.8% of the world’s population (Holden et al., 2016). Although RE develops predominantly during adolescence, its impact persists throughout the lifespan, characterized by various complications (Haarman et al., 2020) that impose substantial disease burdens (Naidoo et al., 2019; Mavi et al., 2022; Zhang et al., 2023; Blind et al., 2021).

Dry eye is a multifactorial chronic ocular surface disease characterized by abnormalities in the quality, quantity, or dynamics of tear production. Inflammatory and immune responses constitute core pathophysiological mechanisms in dry eye, with age, sex, environment, and ocular or systemic diseases serving as significant risk factors (Bron et al., 2017). The global prevalence of dry eye is estimated to be 5%–50% (Stapleton et al., 2017), and is still on the rise.

RE and dry eye are both increasingly recognized as common ocular conditions worldwide. In clinical practice, dry eye is substantially more prevalent among RE patients. Shared pathological mechanisms and risk factors have prompted growing research interest in their putative correlations (Zou et al., 2024; Wu et al., 2025; Ibrahim et al., 2024). Research indicated high comorbidity prevalence between these two conditions, with reported dry eye prevalence of 24.6% in emmetropia, 36.5% in myopia, and 17.4% in hyperopia (Fahmy and Aldarwesh, 2018). Another study found high myopia to be independently associated with an increased risk of symptomatic dry eye (Chen et al., 2024). Potential mechanisms were investigated based on tear film breakup time, higher order aberrations, choroidal thickness, and axial length (Hazra et al., 2022).

Dry eye causes both ocular surface damage and visual function impairment (Wolffsohn et al., 2023), diminishing patients’ quality of life (Cutrupi et al., 2023). Treatment is often delayed due to late presentation after the onset of significant symptoms. Upon diagnosis, long-term management is required (Jones et al., 2017). Nevertheless, effective and widely accessible methods for early screening remain limited. Therefore, screening tools in high-risk populations to identify the subclinical stage of dry eye are urgently needed to guide timely intervention.

Machine learning (ML) is a core branch of artificial intelligence (AI), which has been increasingly integrated into clinical decision support systems in recent years. Ophthalmology has emerged as one of the most advanced fields for AI application, owing to the highly digitized, standardized, and imageable nature of its data. Numerous studies have focused on developing ML models for the automated identification and diagnosis of ocular diseases, covering major conditions affecting the anterior (Elsawy et al., 2021; Wei et al., 2023) and posterior segments of the eye (Chakravarthy et al., 2016; Dai et al., 2024), as well as major blinding diseases like glaucoma (Wu et al., 2022) and cataracts (Lu et al., 2025).

In our earlier studies, we developed ML-based decision support systems for ocular metastasis originating from liver cancer (Sun et al., 2023) and distant metastasis of uveal melanoma (Wu et al., 2024). While ML models excel at extracting nonlinear relationships from complex data (Liu et al., 2023), their complexity often creates the black box phenomenon (Sousa et al., 2024), necessitating rigorous validation (Shi et al., 2023). For the past 2 decades, traditional models such as Gradient-Boosted Decision Trees (GBDT) have dominated the analysis of structured electronic health record data, but has been constrained by challenges in out-of-distribution prediction and cross-dataset knowledge transfer. Hollmann et al. (Hollmann et al., 2025) introduced the Tabular Prior-data Fitted Network (TabPFN) in 2025, a foundational model for small to medium-sized tabular datasets. The key innovation of TabPFN is in-context learning paradigm for tabular data. Pre-trained on millions of synthetic datasets, TabPFN performs inference directly on new data without retraining. This architecture enables remarkable speed, delivering predictions in just 2.8 s. Besides, TabPFN has also demonstrated exceptional performance on medical benchmarks from Open Machine Learning (OpenML), achieving a specificity and accuracy of 0.945 on datasets with up to 500 features, significantly outperforming traditional models like GBDT.

**FIGURE 1:**
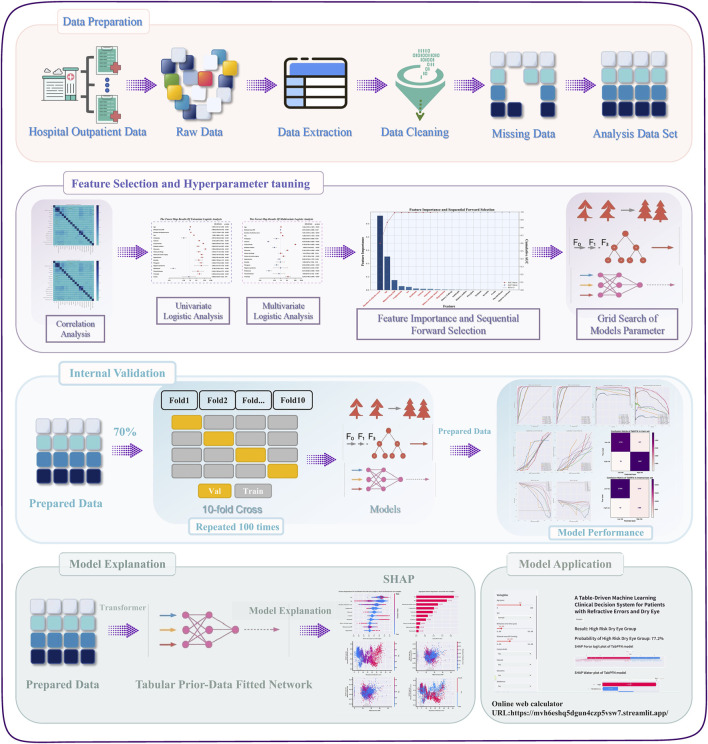
The schematic diagram of the current research work and the corresponding abstract of the research.

Given this context, we developed and evaluated a diagnostic prediction model for estimating the probability of dry eye comorbidity in RE patients based on extensive real-world data from Xiamen Eye Center. Figure 1 illustrates the workflow of our current study. Our work aims to identify key risk factors for dry eye comorbidity and to develop a diagnostic prediction model that serves as a practical clinical tool for estimating the probability of dry eye comorbidity in patients with RE, thereby aiding opportunistic screening and informing targeted clinical decisions.

## 2 Materials and methods

### 2.1 Data source

**FIGURE 2:**
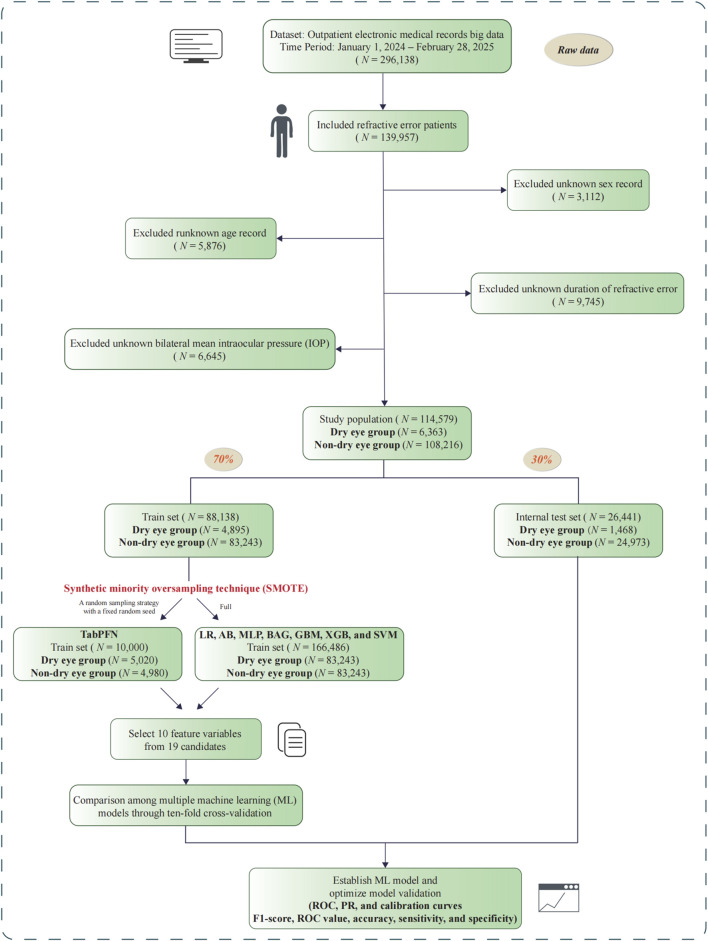
Flowchart of data cleaning process and ML-based portfolio construction in the outpatient database.

We collected data from the outpatient database of Xiamen Eye Center from 1st January 2024 to 28th February 2025. RE and dry eye were identified using the International Classification of Diseases, Tenth Revision codes (ICD-10) H52.7 and H04.12, respectively, based on ophthalmologist-confirmed diagnoses recorded in the medical system. RE diagnosis was based on criteria including either patient self-report or visual acuity examinations combined with optometric results obtained under relaxed accommodation. In our clinical practice, physicians commonly document the broader diagnostic category “Refractive Error” in the electronic health records. Therefore, RE in this study includes myopia, hyperopia, and astigmatism. The diagnosis of dry eye followed the Chinese Dry Eye Diagnostic Consensus (Cornea Group of Ophthalmology Branch of Chinese Medical Association, 2024), a nationally recognized guideline developed with reference to international standards such as the TFOS DEWS framework (Wolffsohn et al., 2017). The unit of analysis was the individual patient. Data were extracted from records categorized under the “First Visit” template. All records were then verified using each patient’s unique identification number, and any subsequent duplicate documentation was removed to minimize potential human errors. Participants with missing data in key features, including sex, age, duration of RE, and intraocular pressure (IOP), were identified as invalid clinical encounters and were excluded during the initial data quality control phase. Specifically, exclusions comprised 3,112 records with unknown sex, 5,876 with unknown age, 9,745 with unknown duration of RE, and 6,645 with unknown IOP, totaling 25,378 exclusions. The corresponding study workflow is illustrated in Figure 2. In total, 114,579 subjects were included in the final analysis. We randomly divided these subjects into the training set and the internal test set at a 7:3 ratio, a conventional split in the field (Sidey-Gibbons and Sidey-Gibbons, 2019). This study was performed in accordance with the tenets of the Declaration of Helsinki and was approved by the Medical Ethics Committee of Xiamen Eye Center and the approved number was XMYKZX-KY-2025–077.

### 2.2 Data selection

Medical records of all participants were collected to acquire basic information about their age, sex, and pathological ocular conditions, including duration of RE, bilateral mean IOP, and other combined ocular and systemic diseases, including glaucoma, cataract, pterygium, uveitis, strabismus, amblyopia, keratitis, conjunctivitis, trichiasis, hypertension, diabetes mellitus, Sjögren’s syndrome, history of allergy, thyroid disease, and history of ocular surgery. Specifically, the above information was extracted from the sections documenting the present illness history and past medical history in the electronic medical records. Data for RE and dry eye variables were extracted from the diagnostic section. Age was stratified into three groups according to the World Health Organization (WHO) report: children (≤17 years), working-age adults (18–59 years), and older adults (≥60 years) (World Health Organization, 2022). None of the mentioned variables contained missing values. A comprehensive description of all 19 input features, including their data types, units of measurement, and specific diagnostic criteria, is provided in Supplementary Table S1.

### 2.3 Data preprocessing and feature screening

*Spearman* correlation analysis was conducted to evaluate the correlation coefficients of these variables in both the training set and the internal test set. Univariate and multivariate LR analyses were subsequently performed on the training set, using dry eye as the outcome variable. Then we determined the final set of feature variables for the ML model by utilizing the feature importance ranking from a random forest (RF) ML algorithm and the sequential forward selection technique (Liu et al., 2012; Marcano-Cedeo et al., 2010). Specifically, we used dry eye as the dependent variable and other feature variables as independent variables to perform RF feature importance ranking. Next, we conducted sequential forward feature selection based on area under the receiver operating characteristic curve (ROC AUC) values, and determined the final set of feature variables to be included in the ML model when the ROC AUC value reached its maximum and the addition of extra feature variables did not result in a significant improvement.

### 2.4 Model construction

Based on the feature variable selection method mentioned earlier, we constructed eight different ML algorithms, including LR (Zhao et al., 2021), adaptive boosting (AB) (Fei et al., 2023), multilayer perceptron (MLP) (Tang et al., 2016), bootstrap aggregating (BAG) (Shi et al., 2022), gradient boosting machine (GBM) (Li et al., 2022), extreme gradient boosting (XGB) (Bakasa and Viriri, 2023), tabular prior-data fitted network (TabPFN) (Hollmann et al., 2025), and support vector machine (SVM) (Silagyi and Liu, 2023). We performed ten-fold cross-validation for the training set data using these eight different ML algorithms. The ROC curve, precision-recall (PR) curve, calibration curve, decision curve analysis (DCA) curve, and corresponding confusion matrix results were evaluated for both the training set and the internal test set. PR curve analysis was introduced to evaluate model performance for imbalanced data (Fu et al., 2019). We also used ROC AUC and PR AUC to evaluate the metrics of each model. Finally, the ML model with the best predictive performance was selected, and a corresponding web calculator was developed. A default probability threshold of 0.5 was applied for classification, which is consistent with common practices in medical ML research (Lyu et al., 2023; Wu et al., 2023).

### 2.5 Model interpretation

SHapley Additive exPlanations (SHAP) is a widely used framework for interpreting black-box ML models based on cooperative game theory (Rodriguez-Perez and Bajorath, 2020). This method allows for the quantitative analysis of the specific contribution values of different feature variables to the occurrence of dry eye in patients with RE. In the SHAP analysis, a positive SHAP value or the color red represents a risk factor for dry eye in patients with RE, while a negative SHAP value or the color blue represents a protective factor (Liu et al., 2023). The optimal model was employed for predicting concurrent dry eye comorbidity in patients with RE, and the importance of the features was ranked from high to low. Interaction effects among key risk factors were analyzed using partial dependence plots (PDP).

### 2.6 Statistical analysis

We conducted data analysis using Python (version 3.8) and R (version 4.3.3). The construction of ML models and hyperparameter tuning were performed on the training set. The model’s accuracy was evaluated using the internal test set. Due to a significant imbalance in sample sizes between dry eye group and non-dry eye group, we applied the Synthetic Minority Oversampling Technique (SMOTE) (Bunkhumpornpat et al., 2024), which was performed exclusively on the training set after the 7:3 data split. To accommodate the architectural constraints of TabPFN, a balanced subset of 10,000 samples was constructed using stratified random sampling with a fixed random seed. The configuration was as follows: k-neighbors = 5, sampling strategy = auto, and random seed = 50. The key code and primary library calls necessary for reproducibility have been made publicly available on GitHub (link: https://github.com/CurryevtWSN/Dry-Eye/tree/main). Hyperparameter optimization was performed using ten-fold cross-validation within the training set, where the validation set comprised 10% of the total training set in each cross-validation cycle, thereby enabling real-time tracking of the model’s status and convergence throughout the training process. For all models, this procedure was performed after the application of SMOTE. The internal test set was left unaltered to maintain the real distribution of data. 95% confidence intervals (CIs) for all key performance metrics, including F1-score, AUC value, accuracy, sensitivity, specificity, Brier score, slope, and intercept were calculated using the bootstrap method (with 1,000 iterations). For continuous numerical data, a normality test was initially conducted. Data conforming to a normal distribution were analyzed using an independent sample *t*-test, expressed as mean ± standard deviation (mean ± SD). Data not conforming to a normal distribution underwent the Mann-Whitney *U* test, expressed as median (Q1, Q3). Chi-squared tests were used to compare categorical variables between groups. In Python, the main libraries used for building ML models and web calculators included sklearn (version 1.2.2), shap (version 0.42.1), tabpfn-extensions (version 2.0), and streamlit (version 1.24.1).

## 3 Results

### 3.1 Subject baseline information and correlation analysis

**FIGURE 3:**
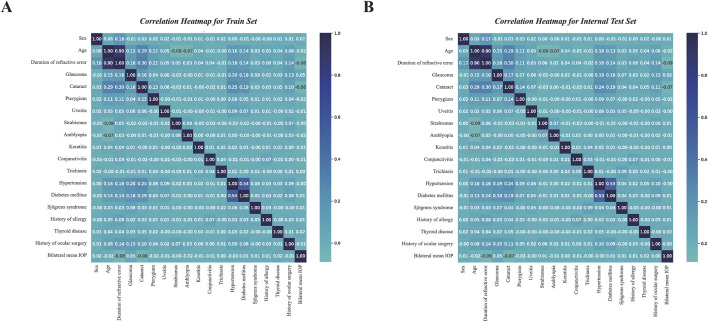
Correlation analysis heatmap among feature variables in the training set **(A)** and internal test set **(B)**. Correlation analysis was conducted using *Spearman* correlation, where darker colors indicate stronger correlations between the feature variables.

We included a total of 114,579 patients diagnosed with RE, randomly dividing them into the training set (n = 88,138) and the internal test set (n = 26,441) in a 7:3 ratio. The comparison of baseline data between the training set and the internal test set showed no differences (*p* > 0.05) (Table 1). The comparison of baseline data between dry eye subgroups in subjects with RE indicated significant differences in age, duration of RE, bilateral mean IOP, sex, ocular abnormalities including glaucoma, cataract, pterygium, strabismus, amblyopia, keratitis, conjunctivitis, and trichiasis, as well as systemic diseases including hypertension, diabetes, Sjögren’s syndrome, history of allergy, thyroid disease, and history of ocular surgery (*p* < 0.001) (Table 2), indicating a strong comorbidity relationship between these two diseases. In the training set, significant differences were observed between dry eye group and non-dry eye group, in terms of age, duration of RE, bilateral mean IOP, sex, glaucoma, cataract, pterygium, strabismus, amblyopia, keratitis, conjunctivitis, trichiasis, hypertension, diabetes, Sjögren’s syndrome, history of allergy, thyroid disease, and history of ocular surgery (*p* < 0.001). In the internal test set, significant differences were also found between the groups with and without dry eye comorbidity, in age, duration of RE, bilateral mean IOP, sex, glaucoma, cataract, pterygium, strabismus, amblyopia, keratitis, conjunctivitis, trichiasis, hypertension, diabetes, Sjögren’s syndrome, history of allergy, thyroid disease, and history of ocular surgery (*p* < 0.001) (Table 3). Furthermore, the *Spearman* correlation analysis of various indicators in both groups was presented in Figure 3. Each cell in the heatmaps represents the *Spearman* correlation coefficient (R), which ranged from −1 to 1. Darker colors signified higher absolute values of R, indicating stronger correlations between indicators. Notably, age demonstrated a strong positive correlation with RE duration (R = 0.90).

**TABLE 1:** Comparison of baseline data between the training set and internal test set.

Variable	Total	Train group	Test group	Statistic	*p*-value
	(N = 114579)	(N = 88138)	(N = 26441)		
Age (years)	17.91 ± 15.20	17.95 ± 15.25	17.80 ± 15.03	−1.397	0.162
Duration of refractive error	7.29 ± 6.70	7.31 ± 6.72	7.24 ± 6.64	−1.424	0.154
Bilateral mean IOP	15.10 ± 3.21	15.10 ± 3.21	15.12 ± 3.19	1.287	0.198
Sex	​	​	​	0.484	0.487
Male	59424(51.86)	45761(51.92)	13663(51.67)	​	​
Female	55155(48.14)	42377(48.08)	12778(48.33)	​	​
Glaucoma	​	​	​	0.001	0.979
No	113101(98.71)	87002(98.71)	26099(98.71)	​	​
Yes	1478(1.29)	1136(1.29)	342(1.29)	​	​
Cataract	​	​	​	0.008	0.928
No	110632(96.56)	85099(96.55)	25533(96.57)	​	​
Yes	3947(3.44)	3039(3.45)	908(3.43)	​	​
Pterygium	​	​	​	0.052	0.819
No	114019(99.51)	87710(99.51)	26309(99.50)	​	​
Yes	560(0.49)	428(0.49)	132(0.50)	​	​
Uveitis	​	​	​	0.088	0.767
No	114419(99.86)	88017(99.86)	26402(99.85)	​	​
Yes	160(0.14)	121(0.14)	39(0.15)	​	​
Strabismus	​	​	​	0.063	0.802
No	109478(95.55)	84222(95.56)	25256(95.52)	​	​
Yes	5101(4.45)	3916(4.44)	1185(4.48)	​	​
Amblyopia	​	​	​	0.219	0.64
No	112659(98.32)	86652(98.31)	26007(98.36)	​	​
Yes	1920(1.68)	1486(1.69)	434(1.64)	​	​
Keratitis	​	​	​	0.016	0.898
No	114396(99.84)	87996(99.84)	26400(99.84)	​	​
Yes	183(0.16)	142(0.16)	41(0.16)	​	​
Conjunctivitis	​	​	​	1.823	0.177
No	109861(95.88)	84470(95.84)	25391(96.03)	​	​
Yes	4718(4.12)	3668(4.16)	1050(3.97)	​	​
Trichiasis	​	​	​	0.14	0.708
No	114038(99.53)	87726(99.53)	26312(99.51)	​	​
Yes	541(0.47)	412(0.47)	129(0.49)	​	​
Hypertension	​	​	​	0.381	0.537
No	113359(98.94)	87190(98.92)	26169(98.97)	​	​
Yes	1220(1.06)	948(1.08)	272(1.03)	​	​
Diabetes mellitus	​	​	​	0.34	0.56
No	113621(99.16)	87393(99.15)	26228(99.19)	​	​
Yes	958(0.84)	745(0.85)	213(0.81)	​	​
Sjögren’s syndrome	​	​	​	0.217	0.641
No	114532(99.96)	88100(99.96)	26432(99.97)	​	​
Yes	47(0.04)	38(0.04)	9(0.03)	​	​
History of allergy	​	​	​	0.076	0.783
No	113888(99.40)	87610(99.40)	26278(99.38)	​	​
Yes	691(0.60)	528(0.60)	163(0.62)	​	​
Thyroid disease	​	​	​	0.308	0.579
No	114491(99.92)	88073(99.93)	26418(99.91)	​	​
Yes	88(0.08)	65(0.07)	23(0.09)	​	​
History of ocular surgery	​	​	​	1.116	0.291
No	113317(98.90)	87151(98.88)	26166(98.96)	​	​
Yes	1262(1.10)	987(1.12)	275(1.04)	​	​
Dry eye	​	​	​	0	1
No	108216(94.45)	83243(94.45)	24973(94.45)	​	​
Yes	6363(5.55)	4895(5.55)	1468(5.55)	​	​

**TABLE 2:** Baseline characteristics of dry eye subgroups in subjects with refractive error.

Variable	Total	Non-dry eye group	Dry eye group	Statistic	*p*-value
	(N = 114579)	(N = 108216)	(N = 6363)		
Age (years)	17.91 ± 15.20	16.87 ± 14.35	35.60 ± 18.02	−81.383	<0.001
Duration of refractive error	7.29 ± 6.70	6.84 ± 6.44	14.91 ± 6.49	−96.379	<0.001
Bilateral mean IOP	15.10 ± 3.21	15.12 ± 3.17	14.78 ± 3.67	7.291	<0.001
Sex	​	​	​	298.788	<0.001
Male	59424(51.86)	56794(52.48)	2630(41.33)	​	​
Female	55155(48.14)	51422(47.52)	3733(58.67)	​	​
Glaucoma	​	​	​	927.594	<0.001
No	113101(98.71)	107087(98.96)	6014(94.52)	​	​
Yes	1478(1.29)	1129(1.04)	349(5.48)	​	​
Cataract	​	​	​	484.837	<0.001
No	110632(96.56)	104800(96.84)	5832(91.65)	​	​
Yes	3947(3.44)	3416(3.16)	531(8.35)	​	​
Pterygium	​	​	​	128.988	<0.001
No	114019(99.51)	107749(99.57)	6270(98.54)	​	​
Yes	560(0.49)	467(0.43)	93(1.46)	​	​
Uveitis	​	​	​	0.018	0.894
No	114419(99.86)	108064(99.86)	6355(99.87)	​	​
Yes	160(0.14)	152(0.14)	8(0.13)	​	​
Strabismus	​	​	​	213.789	<0.001
No	109478(95.55)	103164(95.33)	6314(99.23)	​	​
Yes	5101(4.45)	5052(4.67)	49(0.77)	​	​
Amblyopia	​	​	​	25.375	<0.001
No	112659(98.32)	106352(98.28)	6307(99.12)	​	​
Yes	1920(1.68)	1864(1.72)	56(0.88)	​	​
Keratitis	​	​	​	21.45	<0.001
No	114396(99.84)	108058(99.85)	6338(99.61)	​	​
Yes	183(0.16)	158(0.15)	25(0.39)	​	​
Conjunctivitis	​	​	​	1622.678	<0.001
No	109861(95.88)	104381(96.46)	5480(86.12)	​	​
Yes	4718(4.12)	3835(3.54)	883(13.88)	​	​
Trichiasis	​	​	​	76.419	<0.001
No	114038(99.53)	107752(99.57)	6286(98.79)	​	​
Yes	541(0.47)	464(0.43)	77(1.21)	​	​
Hypertension	​	​	​	358.966	<0.001
No	113359(98.94)	107215(99.07)	6144(96.56)	​	​
Yes	1220(1.06)	1001(0.93)	219(3.44)	​	​
Diabetes mellitus	​	​	​	88.216	<0.001
No	113621(99.16)	107378(99.23)	6243(98.11)	​	​
Yes	958(0.84)	838(0.77)	120(1.89)	​	​
Sjögren’s syndrome	​	​	​	9.704	<0.001
No	114532(99.96)	108177(99.96)	6355(99.87)	​	​
Yes	47(0.04)	39(0.04)	8(0.13)	​	​
History of allergy	​	​	​	84.357	<0.001
No	113888(99.40)	107619(99.45)	6269(98.52)	​	​
Yes	691(0.60)	597(0.55)	94(1.48)	​	​
Thyroid disease	​	​	​	6.831	<0.001
No	114491(99.92)	108139(99.93)	6352(99.83)	​	​
Yes	88(0.08)	77(0.07)	11(0.17)	​	​
History of ocular surgery	​	​	​	345.619	<0.001
No	113317(98.90)	107175(99.04)	6142(96.53)	​	​
Yes	1262(1.10)	1041(0.96)	221(3.47)	​	​

**TABLE 3:** Comparison of baseline data for dry eye comorbidity between the training set and internal test set.

Variable	Total	Test group		Train group		Statistic	*p*-value
	(N = 114579)	Non-dry eye group	Dry eye group	Non-dry eye group	Dry eye group		
		(N = 24973)	(N = 1468)	(N = 83243)	(N = 4895)		
Age	17.91 ± 15.20	16.78 ± 14.19	35.21 ± 17.95	16.90 ± 14.40	35.72 ± 18.04	3306.354	<0.001
Duration of refractive error	7.29 ± 6.70	6.80 ± 6.38	14.76 ± 6.53	6.86 ± 6.46	14.96 ± 6.48	3145.79	<0.001
Bilateral mean IOP	15.10 ± 3.21	15.15 ± 3.17	14.75 ± 3.54	15.11 ± 3.18	14.79 ± 3.71	23.704	<0.001
Sex	​	​	​	​	​	300.272	<0.001
Male	59424(51.86)	13047(52.24)	616(41.96)	43747(52.55)	2014(41.14)	​	​
Female	55155(48.14)	11926(47.76)	852(58.04)	39496(47.45)	2881(58.86)	​	​
Glaucoma	​	​	​	​	​	931.107	<0.001
No	113101(98.71)	24711(98.95)	1388(94.55)	82376(98.96)	4626(94.50)	​	​
Yes	1478(1.29)	262(1.05)	80(5.45)	867(1.04)	269(5.50)	​	​
Cataract	​	​	​	​	​	487.016	<0.001
No	110632(96.56)	24192(96.87)	1341(91.35)	80608(96.83)	4491(91.75)	​	​
Yes	3947(3.44)	781(3.13)	127(8.65)	2635(3.17)	404(8.25)	​	​
Pterygium	​	​	​	​	​	133.39	<0.001
No	114019(99.51)	24866(99.57)	1443(98.30)	82883(99.57)	4827(98.61)	​	​
Yes	560(0.49)	107(0.43)	25(1.70)	360(0.43)	68(1.39)	​	​
Uveitis	​	​	​	​	​	0.247	0.97
No	114419(99.86)	24936(99.85)	1466(99.86)	83128(99.86)	4889(99.88)	​	​
Yes	160(0.14)	37(0.15)	2(0.14)	115(0.14)	6(0.12)	​	​
Strabismus	​	​	​	​	​	214.842	<0.001
No	109478(95.55)	23798(95.29)	1458(99.32)	79366(95.34)	4856(99.20)	​	​
Yes	5101(4.45)	1175(4.71)	10(0.68)	3877(4.66)	39(0.80)	​	​
Amblyopia	​	​	​	​	​	26.149	<0.001
No	112659(98.32)	24552(98.31)	1455(99.11)	81800(98.27)	4852(99.12)	​	​
Yes	1920(1.68)	421(1.69)	13(0.89)	1443(1.73)	43(0.88)	​	​
Keratitis	​	​	​	​	​	24.014	<0.001
No	114396(99.84)	24939(99.86)	1461(99.52)	83119(99.85)	4877(99.63)	​	​
Yes	183(0.16)	34(0.14)	7(0.48)	124(0.15)	18(0.37)	​	​
Conjunctivitis	​	​	​	​	​	1632.199	<0.001
No	109861(95.88)	24110(96.54)	1281(87.26)	80271(96.43)	4199(85.78)	​	​
Yes	4718(4.12)	863(3.46)	187(12.74)	2972(3.57)	696(14.22)	​	​
Trichiasis	​	​	​	​	​	78.455	<0.001
No	114038(99.53)	24863(99.56)	1449(98.71)	82889(99.57)	4837(98.82)	​	​
Yes	541(0.47)	110(0.44)	19(1.29)	354(0.43)	58(1.18)	​	​
Hypertension	​	​	​	​	​	362.13	<0.001
No	113359(98.94)	24749(99.10)	1420(96.73)	82466(99.07)	4724(96.51)	​	​
Yes	1220(1.06)	224(0.90)	48(3.27)	777(0.93)	171(3.49)	​	​
Diabetes mellitus	​	​	​	​	​	90.111	<0.001
No	113621(99.16)	24786(99.25)	1442(98.23)	82592(99.22)	4801(98.08)	​	​
Yes	958(0.84)	187(0.75)	26(1.77)	651(0.78)	94(1.92)	​	​
Sjögren’s syndrome	​	​	​	​	​	13.461	<0.001
No	114532(99.96)	24965(99.97)	1467(99.93)	83212(99.96)	4888(99.86)	​	​
Yes	47(0.04)	8(0.03)	1(0.07)	31(0.04)	7(0.14)	​	​
History of allergy	​	​	​	​	​	86.552	<0.001
No	113888(99.40)	24830(99.43)	1448(98.64)	82789(99.45)	4821(98.49)	​	​
Yes	691(0.60)	143(0.57)	20(1.36)	454(0.55)	74(1.51)	​	​
Thyroid disease	​	​	​	​	​	9.144	0.027
No	114491(99.92)	24952(99.92)	1466(99.86)	83187(99.93)	4886(99.82)	​	​
Yes	88(0.08)	21(0.08)	2(0.14)	56(0.07)	9(0.18)	​	​
History of ocular surgery	​	​	​	​	​	354.498	<0.001
No	113317(98.90)	24756(99.13)	1410(96.05)	82419(99.01)	4732(96.67)	​	​
Yes	1262(1.10)	217(0.87)	58(3.95)	824(0.99)	163(3.33)	​	​

### 3.2 Univariate and multivariate logistic regression (LR)

**FIGURE 4:**
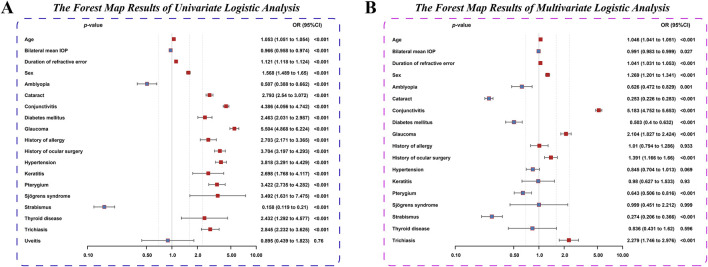
Forest plot of univariate **(A)** and multivariate **(B)** logistic regression analyses for dry eye comorbidity in patients with refractive error. In the figure, blue indicates protective factors (OR and 95% CI all less than 1), while red indicates risk factors (OR and 95% CI all greater than 1).

In the univariate LR analysis, factors such as older age, longer duration of RE, female sex, and the presence of conjunctivitis, glaucoma, cataract, diabetes mellitus, hypertension, keratitis, pterygium, Sjogren’s syndrome, thyroid disease, trichiasis, and history of ocular surgery were identified as risk factors for dry eye comorbidity [odds ratio (OR) and 95% CI > 1, *p* < 0.001]. Conversely, higher bilateral mean IOP, the presence of amblyopia and strabismus were identified as protective factors (OR and 95% CI < 1, *p* < 0.001). In the multivariate LR analysis, the results showed that, older age, longer duration of RE, female sex, the presence of conjunctivitis, glaucoma, trichiasis, and history of ocular surgery were identified as independent risk factors for dry eye comorbidity (OR and 95% CI > 1, *p* < 0.001), while the presence of amblyopia, cataract, pterygium, strabismus, and diabetes mellitus were identified as protective factors (OR and 95% CI < 1, *p* < 0.001) (Table 4). Regarding age, both working-age adults and older adults were identified as significant risk factors in univariate and multivariate LR analyses. The univariate analysis indicated that both working-age adults (OR: 11.04, 95% CI: 10.33–11.81) and older adults (OR: 18.31, 95% CI: 16.57–20.23) were at significantly higher risk for dry eye comorbidity (both *p* < 0.001), with a notably greater risk observed among older adults relative to working-age adults. In the multivariate analysis, the associations remained statistically significant (both *p* < 0.001). The adjusted OR for working-age adults was 5.70 (95% CI: 5.21–6.23), while that for older adults was 6.96 (95% CI: 5.98–8.11), further confirming the graded increase in risk across age groups. Forest plots illustrating statistically significant indicators corresponding to the results of univariate and multivariate LR analyses are presented in Figure 4.

**TABLE 4:** Univariate and multivariate logistic regression results for patients with refractive error.

Variable	Factor	Univariate logistic analysis	*p*-value	Multivariate logistic analysis	*p*-value
		OR (95% CI)		OR (95% CI)	
Bilateral mean IOP	—	0.97 (0.96–0.97)	<0.001	0.99 (0.98–1.00)	0.027
Refractive error time	—	1.12 (1.12–1.12)	<0.001	1.04 (1.03–1.05)	<0.001
Age (years)	Children	Ref	Ref	Ref	Ref
	Working-age adults	11.04 (10.33–11.81)	<0.001	5.70 (5.21–6.23)	<0.001
	Older adults	18.31 (16.57–20.23)	<0.001	6.96 (5.98–8.11)	<0.001
Sex	Male	Ref	Ref	Ref	Ref
	Female	1.57 (1.49–1.7)	<0.001	1.27 (1.20–1.34)	<0.001
Amblyopia	No	Ref	Ref	Ref	Ref
	Yes	0.51 (0.39–0.66)	<0.001	0.63 (0.47–0.83)	0.001
Cataract	No	Ref	Ref	Ref	Ref
	Yes	2.79 (2.54–3.07)	<0.001	0.253 (0.23–0.28)	<0.001
Conjunctivitis	No	Ref	Ref	Ref	Ref
	Yes	4.39 (4.06–4.74)	<0.001	5.18 (4.75–5.65)	<0.001
Diabetes mellitus	No	Ref	Ref	Ref	Ref
	Yes	2.46 (2.03–2.99)	<0.001	0.50 (0.40–0.63)	<0.001
Glaucoma	No	Ref	Ref	Ref	Ref
	Yes	5.50 (4.87–6.22)	<0.001	2.10 (1.83–2.42)	<0.001
History of allergy	No	Ref	Ref	Ref	Ref
	Yes	2.70 (2.17–3.37)	<0.001	1.01 (0.79–1.29)	0.933
History of ocular surgery	No	Ref	Ref	Ref	Ref
	Yes	3.70 (3.20–4.30)	<0.001	1.39 (1.17–1.66)	<0.001
Hypertension	No	Ref	Ref	Ref	Ref
	Yes	3.82 (3.29–4.43)	<0.001	0.85 (0.70–1.01)	0.069
Keratitis	No	Ref	Ref	Ref	Ref
	Yes	2.70 (1.77–4.12)	<0.001	0.98 (0.63–1.53)	0.93
Pterygium	No	Ref	Ref	Ref	Ref
	Yes	3.42 (2.74–4.28)	<0.001	0.64 (0.51–0.82)	<0.001
Sjögren’s syndrome	No	Ref	Ref	Ref	Ref
	Yes	3.49 (1.63–7.48)	0.001	1.00 (0.45–2.21)	0.999
Strabismus	No	Ref	Ref	Ref	Ref
	Yes	0.16 (0.12–0.21)	<0.001	0.27 (0.21–0.37)	<0.001
Thyroid disease	No	Ref	Ref	Ref	Ref
	Yes	2.43 (1.29–4.58)	0.006	0.84 (0.43–1.62)	0.596
Trichiasis	No	Ref	Ref	Ref	Ref
	Yes	2.85 (2.23–3.63)	<0.001	2.28 (1.75–2.98)	<0.001
Uveitis	No	Ref	Ref	Ref	Ref
	Yes	0.90 (0.44–1.82)	0.76	—	—

### 3.3 Model performance

**FIGURE 5:**
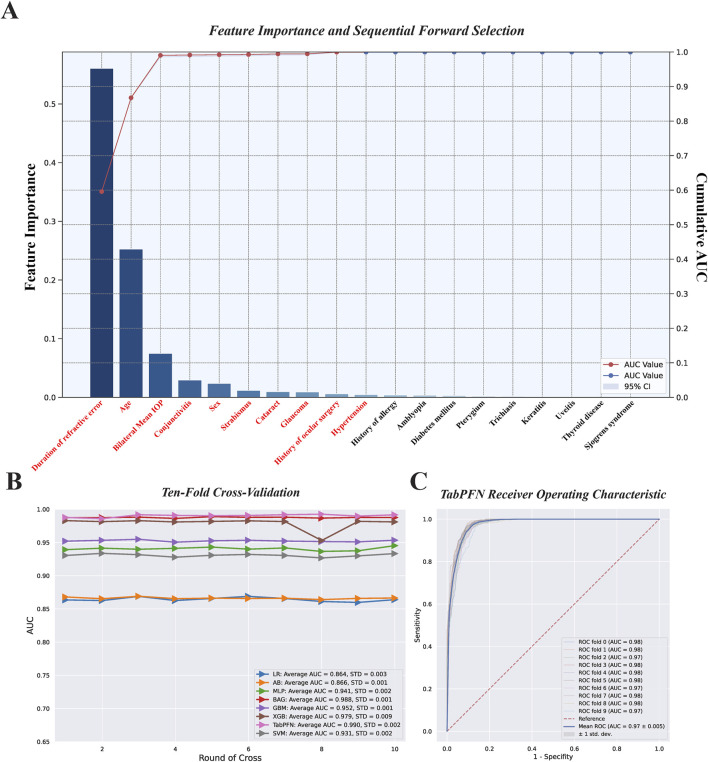
**(A–C)** Sequential forward feature selection based on the random forest method and ten-fold cross-validation results on the training set.

Based on the forward sequence feature selection method, the selected feature variables for inclusion in the ML model include duration of RE, age, bilateral mean IOP, conjunctivitis, sex, strabismus, cataract, glaucoma, history of ocular surgery, and hypertension (Figure 5A). The ten-fold cross-validation results for the training set, after performing SMOTE oversampling, showed that TabPFN had the best area under the curve (AUC) value. The average AUC was 0.990 with a standard deviation (SD) of 0.002 (Figure 5B). For the optimal TabPFN method, we separately plotted the ROC curve for ten-fold cross-validation without SMOTE-balanced data, with an average AUC of 0.97 and a SD of 0.005 (Figure 5C).

**FIGURE 6:**
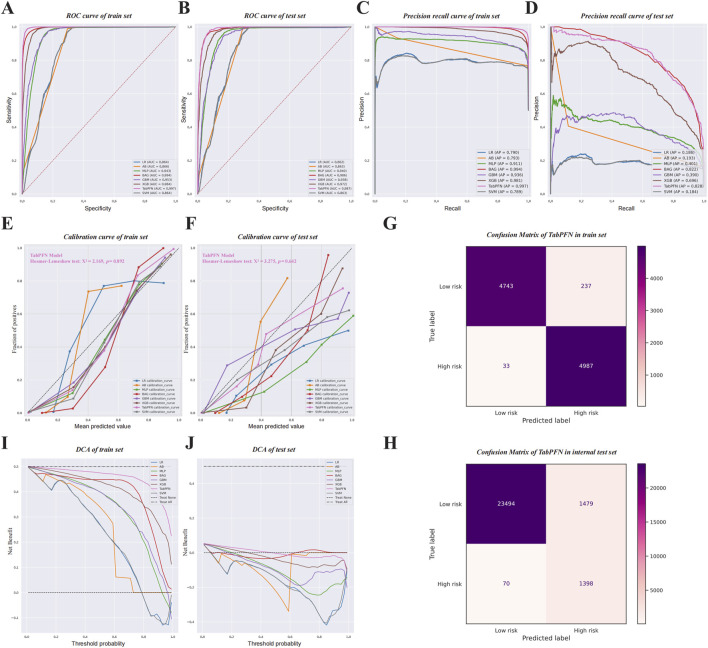
ROC curves **(A,B)**, PR curves **(C,D)**, calibration curves **(E,F)**, and DCA curves **(I, J)** for the training set **(A,C,E,I)** and internal test set **(B,D,F, J)**, along with the confusion matrix results **(G,H)** for the best ML model.

**FIGURE 7:**
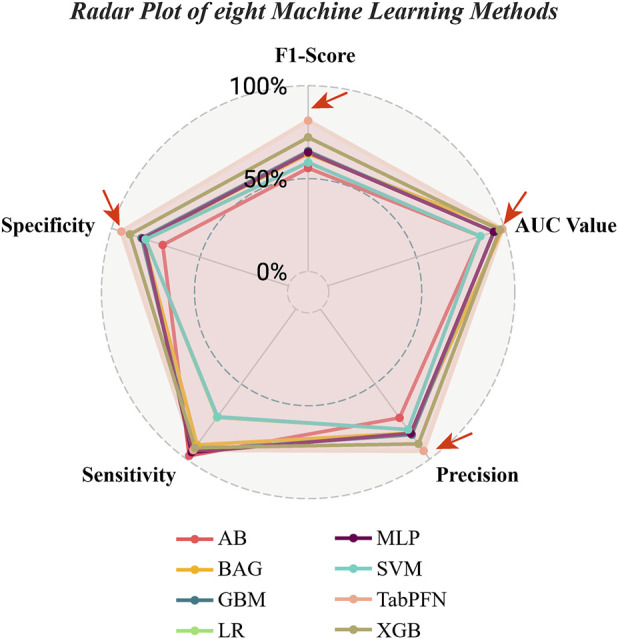
Radar chart evaluating performance metrics for eight different machine learning methods. The TabPFN model has the highest values in F1 score, AUC value, specificity, and precision.

In the training set and internal test set, the TabPFN model performed the best, with corresponding ROC AUC values of 0.997 and 0.987 (Figures 6A,B). The PR curve results showed that the TabPFN model achieved the highest PR AUC values in both training set (0.997) and internal test set (0.828) (Figures 6C,D). Calibration curve results for different ML models in the training set and internal test set also indicated that the TabPFN model had the best fit, with its curve aligning most closely to the ideal diagonal. The Hosmer-Lemeshow test results for TabPFN (χ^2^ = 2.169, *p* = 0.892 for the training set; χ^2^ = 3.275, *p* = 0.642 for the internal test set) indicated no statistically significant deviation between predicted probabilities and observed outcomes, confirming its statistically reliable calibration performance (Figures 6E,F). The relatively low Brier score of TabPFN [0.036 (95% CI: 0.034–0.038)] indicated high accuracy in probability predictions. The calibration slope of 0.926 (95% CI: 0.865–0.993) suggested near-ideal agreement between predicted and observed probabilities. The calibration intercept of −1.908 (95% CI: −1.998 to −1.814) revealed minor risk overestimation, particularly in the lower probability range. Finally, on the balanced training set, the optimal TabPFN model achieved a prediction accuracy of 0.973, while on the internal test set without data balancing, the TabPFN model reached a prediction accuracy of 0.941. On the internal test set, the TabPFN model correctly classified 23,494 low-risk cases and misclassified 1,479 low-risk cases as high risk. Among high-risk cases, it correctly identified 1,398 and misclassified 70 as low risk (Figure 6H). Additionally, it demonstrated a positive predictive value (PPV) of 0.486 and a negative predictive value (NPV) of 0.997 in the test set (Figures 6G,H). DCA demonstrated that the TabPFN model consistently provided the highest net benefit across a wide range of threshold probabilities in both the training set (Figure 6I) and the test set (Figure 6J). Through the comparison of eight different ML methods, we have generated a table presenting corresponding evaluation metrics. The TabPFN method surpasses the other seven models, demonstrating the highest values for F1-score [0.812 (95% CI: 0.804–0.821)], AUC value [0.987 (95% CI: 0.985–0.988)] accuracy [0.941 (95% CI: 0.887–0.957)], and specificity [0.941 (95% CI: 0.938–0.948)]. Specific numerical values are provided in Table 5, and the corresponding visual representation in the form of a radar chart is depicted in Figure 7.

**TABLE 5:** Comparison of performance across machine learning models.

Classifier	F1 (95% CI)	AUC (95% CI)	Accuracy (95% CI)	Sensitivity (95% CI)	Specificity (95% CI)	Brier (95% CI)	Slope (95% CI)	Intercept (95% CI)
AB	0.557 (0.550–0.563)	0.863 (0.859–0.867)	0.725 (0.648–0.758)	0.977 (0.970–0.984)	0.711 (0.705–0.716)	0.127 (0.125–0.129)	2.461 (2.308–2.644)	−2.765 (−2.864 to −2.677)
LR	0.587 (0.580–0.595)	0.862 (0.856–0.868)	0.803 (0.742–0.852)	0.723 (0.699–0.743)	0.807 (0.802–0.812)	0.159 (0.156–0.162)	0.676 (0.650–0.704)	−2.798 (−2.852 to −2.744)
BAG	0.633 (0.621–0.678)	0.986 (0.984–0.987)	0.826 (0.813–0.844)	0.905 (0.864–0.921)	0.821 (0.804–0.861)	0.065 (0.063–0.066)	3.672 (3.475–3.867)	−3.278 (−3.460 to −3.108)
MLP	0.641 (0.642–0.658)	0.940 (0.933–0.947)	0.828 (0.749–0.860)	0.954 (0.942–0.964)	0.821 (0.805–0.835)	0.108 (0.105–0.111)	0.835 (0.792–0.883)	−2.535 (−2.612 to −2.460)
GBM	0.648 (0.641–0.659)	0.938 (0.933–0.942)	0.837 (0.752–0.863)	0.941 (0.933–0.956)	0.830 (0.828–0.838)	0.103 (0.101–0.106)	0.926 (0.875–0.981)	−2.452 (−2.528 to −2.384)
XGB	0.722 (0.718–0.746)	0.972 (0.969–0.975)	0.898 (0.813–0.923)	0.924 (0.903–0.948)	0.896 (0.881–0.908)	0.061 (0.059–0.063)	0.926 (0.867–0.990)	−2.254 (−2.363 to −2.145)
TabPFN	0.812 (0.804–0.821)	0.987 (0.985–0.988)	0.941 (0.887–0.957)	0.952 (0.927–0.980)	0.941 (0.938–0.948)	0.036 (0.034–0.038)	0.926 (0.865–0.993)	−1.908 (−1.998 to −1.814)
SVM	0.587 (0.579–0.594)	0.863 (0.858–0.869)	0.803 (0.741–0.852)	0.718 (0.697–0.741)	0.808 (0.802–0.812)	0.160 (0.156–0.163)	0.688 (0.662–0.717)	−2.800 (−2.853 to −2.746)

To ensure a fair baseline comparison, all eight models were additionally evaluated using the same balanced subset of 10,000 samples, and TabPFN consistently exhibited superior predictive performance. In the ten-fold CV, TabPFN achieved the highest average ROC AUC of 0.990, and an SD of 0.002 (Supplementary Figure S1A). When evaluated on the internal test set, it outperformed the other seven models, yielding an ROC AUC of 0.986 and a PR AUC of 0.827 (Supplementary Figure S1B,C). Additionally, it demonstrated a PPV of 0.945 and a NPV of 0.939 in the test set (Supplementary Figure S1D). Overall, TabPFN demonstrated the most robust discriminatory ability across multiple evaluation metrics, achieving an F1 score of 0.970, accuracy of 0.945, ROC AUC of 0.986, and specificity of 0.503, along with a consistently high sensitivity of 0.996 (Supplementary Table S2).

### 3.4 Model interpretability

**FIGURE 8:**
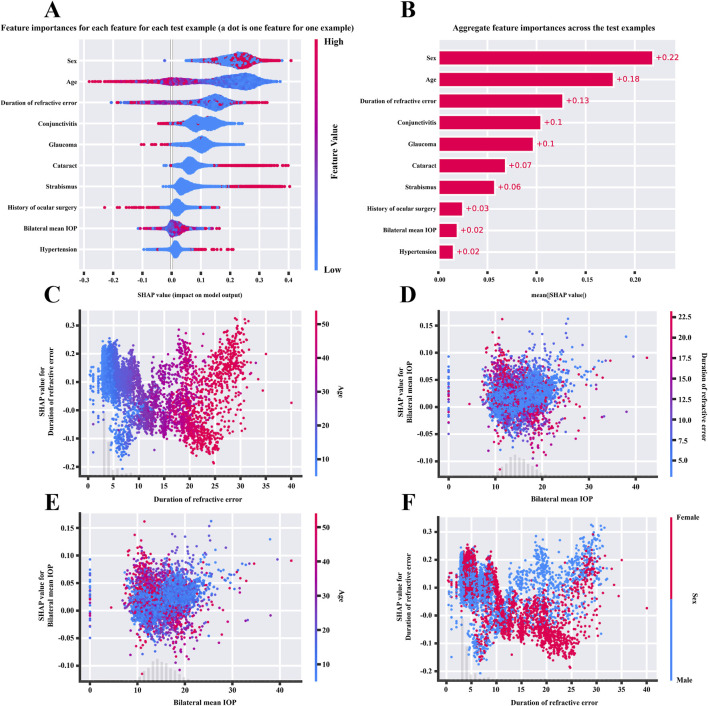
**(A,B)** SHAP model explanation for feature variables based on the TabPFN model. The importance ranking of feature variables for patients with refractive error using the TabPFN model. In these figures, red indicates variables that act as risk factors for dry eye, while blue indicates variables acting as protective factors. **(C–F)** PDP results for duration of refractive error and age, bilateral mean IOP and duration of refractive error, bilateral mean IOP and age, and duration of refractive error and sex.

According to the best ML model, the TabPFN model, we constructed a feature importance ranking based on SHAP. The importance ranking for patients with RE developing dry eye was as follows: sex, age, duration of RE, conjunctivitis, glaucoma, cataract, strabismus, history of ocular surgery, bilateral mean IOP, and hypertension (Figures 8A,B). PDP results were generated to visualize the interaction relationships among the key risk factors (Figures 8C–F). PDP interaction of RE duration and age revealed that a longer duration of RE had a stronger positive impact on model prediction outcomes, especially in the elderly population (Figure 8C). While PDP interaction of duration of RE and sex demonstrated that a longer duration of RE was more predictive of dry eye risk in female patients, as indicated by higher SHAP values (Figure 8F).

### 3.5 Online web calculator

**FIGURE 9:**
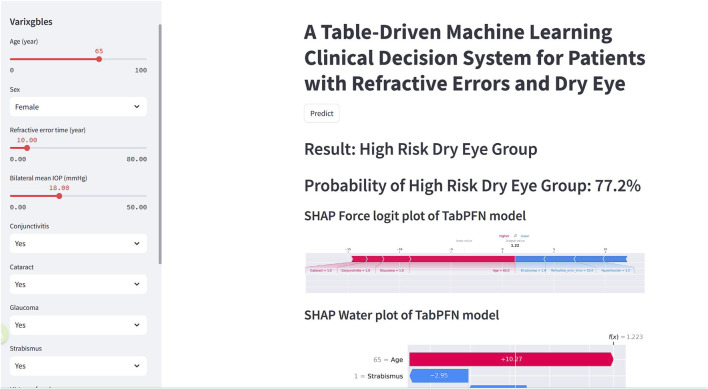
Online web calculator for calculating dry eye risk probability in patients with refractive error.

Based on the optimal TabPFN model, we have developed a web calculator for real-time personalized calculation of the risk probability of dry eye in patients with RE. Users can input specific values for the feature variables on the left side of the web page to calculate the risk probability of dry eye (Figure 9) (URL: https://mvh6eshq5dgun4czp5vsw7.streamlit.app/). Additionally, the tool provides real-time output of each input feature’s contribution, along with an overall feature importance ranking. If poor quality or missing data are detected, the system will withhold the prediction and immediately alert the user to correct the input.

## 4 Discussion

RE and dry eye represent two highly prevalent global ocular public health challenges, both significantly impairing quality of life and imposing long-term economic burdens. A notable degree of comorbidity is observed between the two diseases (Wang et al., 2022). Dry eye causes ocular surface damage and visual dysfunction, which significantly reduces patients’ productivity and wellbeing. However, early prevention and intervention is often challenging as patients typically present after experiencing obvious symptoms. Therefore, early screening of high-risk populations is crucial for timely intervention, potentially reducing disease incidence, progression, and associated societal costs. However, effective and accessible diagnostic prediction models remain lacking.

### 4.1 Superior performance of the TabPFN model

This study leveraged a large-scale real-world clinical dataset to specifically focus on the comorbidity of dry eye in RE patitents. The observed baseline differences suggest that dry eye in RE patients represents a non-random clustering, systematically linked to a distinct demographic and clinical profile. Utilizing only ten key features, TabPFN achieved the best overall performance among 8 ML models for dry eye screening in RE patients. Compared with previously reported ML-based comorbidity prediction models developed for chronic disease populations, including neurodegenerative disease in diabetic patients, depression in individuals with chronic obstructive pulmonary disease (COPD) and asthma, and vascular comorbidities among symptomatic COPD cohorts (Sang et al., 2024; Feng et al., 2025; Nie et al., 2025; Gu et al., 2025), the TabPFN model demonstrated excellent screening performance. In particular, its specificity and accuracy both reached 0.941, exceeding the performance reported in those models. This highlights its superior capability for accurate identification of high-risk individuals. The TabPFN model achieved a high NPV of 0.997 on the test set, indicating a low risk of omission. The PPV of 0.486 was more interpreted as a strategic trade-off to preserve high sensitivity in the context of the low sample prevalence. With robust performance across multiple key metrics, including F1-score, ROC AUC, accuracy, and specificity, TabPFN demonstrates not only strong generalizability but also significant potential as a high-performance screening tool, capable of identifying high-risk cases within complex, real-world data environments.

TabPFN represents a groundbreaking foundational model specifically designed for structured clinical data. Pre-trained on a wide range of tabular tasks, TabPFN has acquired transferable prior knowledge to perform inference directly on new clinical datasets, without requiring complex feature engineering or additional model retraining. The workflow of TabPFN is illustrated in Supplementary Figure S2. TabPFN demonstrates overwhelming superiority on datasets containing fewer than 10,000 samples, reaching optimal results in just 2.8 s with minimal computational resource (Hollmann et al., 2025), thereby enabling rapid deployment in clinical settings.

The RE patient cohort showed substantial class imbalance, with a dry eye to non-dry eye ratio of approximately 1:17. This imbalance represents an inherent characteristic of real-world outpatient data yet poses methodological challenges for predictive modeling. Although SMOTE was applied to improve minority-class learning, the potential risk of overfitting on synthetic samples warrants careful consideration. To mitigate these risks, SMOTE was strictly implemented within the training set only, while the internal test set preserved the original imbalanced distribution, enabling a realistic evaluation of clinical applicability (Bunkhumpornpat et al., 2024). Moreover, we employed PR AUC—a more informative metric than ROC AUC for imbalanced data—to evaluate minority class identification and clinical screening capability. TabPFN achieved a PR AUC of 0.828 on the test set, indicating robust minority-class detection. By utperforming other ML models across key metrics, TabPFN demonstrates genuine clinical generalization rather than an artifact of synthetic oversampling. To address SMOTE’s inherent limitations, future efforts should compare diverse imbalance-handling strategies and conduct multi-center external validation to ensure generalizability.

### 4.2 Clinical significance of key predictor variables

In our study, multivariable LR analysis identified older age, longer RE duration, female sex, conjunctivitis, glaucoma, trichiasis, and history of ocular surgery as independent risk factors, whereas amblyopia, cataract, pterygium, strabismus, and diabetes mellitus showed inverse associations. Importantly, the observed inverse associations may not imply genuine biological protection but could reflect differences in surveillance intensity or residual confounding. For instance, patients with cataract or diabetes may receive more frequent eye examinations and intensive ocular surface care, potentially lowering the likelihood of a concurrent dry eye diagnosis. Additionally, residual confounding and complex interactions between systemic and ocular factors may also attenuate or reverse these associations after multivariable adjustment. Therefore, these findings may be more appropriately viewed as adjusted statistical associations, rather than as direct evidence of causal or protective effects, highlighting the importance of cautious interpretation.

The key predictive factors identified by SHAP analysis in this study, such as age, sex, and duration of RE, exhibit strong statistical associations that align with previously reported epidemiological characteristics of dry eye. This model-driven identification of older age as a key predictor is consistent with global epidemiological data documenting a significant age-related increase in dry eye prevalence (Stapleton et al., 2017). The prominence of female sex as a risk factor also indicated a statistically significant correlation well-supported in the literature (Sullivan et al., 2017). This sex disparity may be due to sex hormone levels, which potentially serve as a key driver underlying the higher dry eye prevalence in women (McKay et al., 2022). The statistical link between a longer duration of RE and dry eye risk, identified in our study, is also consistent with clinical observations. It has been suggested that uncorrected or under-corrected RE may contribute to persistent accommodative strain and asthenopia (Cao et al., 2025), reducing blink rate and increasing tear evaporation, thereby destabilizing the tear film. Furthermore, common methods of refractive correction, such as contact lens wear and refractive surgery, can directly impact ocular surface health (Stapleton et al., 2021; Wen et al., 2017). In addition, conjunctivitis, history of ocular surgery, and history of allergy are also identified as significant predictors of dry eye in patients with RE. These model-derived patterns align with established clinical and research evidence (Akasaki et al., 2022; Chinese Branch of the Asian Dry Eye Society, 2021). The high degree of concordance between the important features identified by our model and the established medical knowledge base offers valuable clues. This alignment motivates further investigation through more rigorously designed studies, including comprehensive basic research and large-scale randomized controlled trials, to confirm the biological vadality of these model-driven associations. In summary, the overall consistency of these statistical associations with existing evidence demonstrates that AI models based on real-world data can effectively integrate traditional knowledge into reliable tools for precise risk assessment.

### 4.3 Major innovations

The innovations of this study are multifaceted. First, the use of large-scale, real-world clinical data effectively captures the complexities of actual clinical practice, enhancing external validity and practical utility of the findings. Second, this research first develops a diagnostic prediction model specifically focused on the comorbidity of RE and dry eye, thereby addressing a significant gap in this specific research area. Methodologically, the study encompasses a complete ML pipeline, from feature engineering, model training and validation, to explainability analysis, ensuring rigorous evaluation of model performance. Furthermore, the online web calculator is designed to be accessible to users without a technical background, including both ophthalmologists and patients. This converts the complex ML model into a user-friendly clinical assistant, effectively promoting the translation of AI into a practical clinical tool.

### 4.4 Clinical application value

The diagnostic prediction model developed in this study offers a practical clinical tool for estimating the probability of dry eye comorbidity in RE patients, holding significant clinical implications and broad application prospects. Primarily, this system facilitates the early identification and intervention for concurrent dry eye in this population, shifting the paradigm from reactive treatment to proactive prevention. Moreover, this system facilitates rapid diagnostic prediction of dry eye comorbidities in RE patients in primary care settings or ophthalmology clinics, identifying high-risk individuals at subclinical or early disease stages for prioritized comprehensive dry eye examinations, thereby enabling early intervention. For patients, the system provides a quick and convenient online prediction tool. By understanding their individual risk, patients can increase their awareness of the disease, adopt better eye care habits, and enhance self-management, potentially reducing the incidence and progression of the disease from the outset. Overall, this diagnostic prediction model supports opportunistic screening and guides targeted clinical decision-making.

### 4.5 Study limitations and future directions

This study also has several limitations. First, despite our large sample size, data from a single ophthalmology center might be subject to a certain degree of selection bias. External validation across diverse geographic regions and populations is necessary to confirm generalizability in the future. Second, due to the nature of retrospective study, our analysis might not fully account for all potential confounding factors. Besides, the current model functions as a diagnostic prediction tool for identifying dry eye comorbidity rather than a prognostic model for predicting future incidence, and the possibility of residual label leakage or co-diagnosis signals can not be ruled out. Strictly designed longitudinal studies with explicitly separated temporal data, particularly those encompassing longer time frames and larger sample sizes, are expected to further validate the model’s efficacy in the future. Additionally, it is important to note that our model utilized structured electronic medical records data and did not incorporate specific tear film, meibomian gland, or ocular surface imaging parameters. Yet, it still yielded excellent predictive results. The integration of these multi-dimensional features in the future represents a promising direction to substantially improve the model’s predictive performance and explanatory power. We found that the subjects with RE were predominantly myopic patients in our study, yet we did not perform subgroup comparison in this analysis, which could be further refined in subsequent studies.

Future research directions could also advance along these two aspects. First, transiting from risk prediction to intervention outcome prediction, providing data-driven recommendations for optimal initial treatment selection. Second, developing dynamic prediction models that incorporate longitudinal follow-up data, such as changes in refraction, medication adherence, or symptom fluctuations, which could continuously update risk assessments using time-series analysis techniques, therefore facilitating personalized and dynamic risk trajectory monitoring.

## 5 Conclusions

In summary, this study developed a high-performance, interpretable ML system based on a large-scale real-world clinical dataset for effcient diagnostic prediction of dry eye risk in patients with RE. The system holds significant potential as a predictive aid for clinical decision-making, enabling more timely and personalized patient management, thereby offering substantial clinical value and promising application prospects.

## Data Availability

The original contributions presented in the study are included in the article/Supplementary Material, further inquiries can be directed to the corresponding authors.
