# Supplementary material for: A TabPFN-based prediction system for refractive error and dry eye comorbidity: a retrospective study using large-scale real-world data
Source: Front Cell Dev Biol. 2026 Apr 9;14:1770427. doi: 10.3389/fcell.2026.1770427 (PMC13102667; doi:10.3389/fcell.2026.1770427)
Supplement: Supplementary file 1 [file DataSheet1.docx]

**Supplementary files**

**Title:** A TabPFN-Based Prediction System for Refractive Error and Dry Eye Comorbidity: A Retrospective Study Using Large-Scale Real-World Data

Author

**Author**

Danyi Qin^1†^, Wenying Guan^1†^, Shinan Wu^1†^, Changsheng Xu^1^, Yuwen Liu^1^, BingYan^1^, Jingyao Lv^1^, Xiaoxin Li^1*^, Zuguo Liu^1, 2*^

^1^Xiamen University affiliated Xiamen Eye Center; Fujian Provincial Key Laboratory of Ophthalmology and Visual Science; Fujian Engineering and Research Center of Eye Regenerative Medicine; Eye Institute of Xiamen University; School of Medicine, Xiamen University, Xiamen, Fujian 361005, China

^2^Department of Ophthalmology, The First Affiliated Hospital of University of South China, Hengyang Medical School, University of South China, Hengyang, Hunan 421001, China

^†^These authors contributed equally to this work.

***Corresponding author.**

**Zuguo Liu, Ph.D., M.D.**

Xiamen University Affiliated Xiamen Eye Center, Fujian Provincial Key Laboratory of Ophthalmology and Visual Science, Fujian Engineering and Research Center of Eye Regenerative Medicine, Eye Institute of Xiamen University, School of Medicine, Xiamen University, Xiamen, Fujian 361005, China

**Email:** zuguoliu@xmu.edu.cn

**Xiaoxin Li, Ph.D., M.D.**

Xiamen University Affiliated Xiamen Eye Center, Fujian Provincial Key Laboratory of Ophthalmology and Visual Science, Fujian Engineering and Research Center of Eye Regenerative Medicine, Eye Institute of Xiamen University, School of Medicine, Xiamen University, Xiamen, Fujian 361005, China

**Email:** dr_lixiaoxin@163.com

| **Table S1. Descriptions of the candidate predictors.** | | | |
| --- | --- | --- | --- |
| **Feature Name** | **Data Type** | **Units / Encoding** | **Definition / Measurement Method** |
| **Demographics** |  |  |  |
| Age | Categorical | 000 = Children, 010 = Working-age adults, 100 = Older adults | Calculated based on the date of birth and the date of first visit (Children: ≤17 years; Working-age adults: 18-59 years; Older adults as ≥60 years) |
| Sex | Categorical | 0 = Female, 1 = Male | Biological sex extracted from electronic health records |
| Ocular Parameters |  |  |  |
| Bilateral mean IOP | Continuous | mmHg | The average IOP of both eyes, measured using non-contact tonometry. |
| Duration of refractive error | Continuous | Years | The duration since the initial diagnosis of refractive error self-reported by the patient |
| **Ocular History & Comorbidities** |  |  |  |
| Amblyopia | Binary | 0 = No, 1 = Yes | Defined by ICD-10 code H53.0 |
| Cataract | Binary | 0 = No, 1 = Yes | Defined by ICD-10 code H25/H26 |
| Conjunctivitis | Binary | 0 = No, 1 = Yes | Defined by ICD-10 code H10 |
| Glaucoma | Binary | 0 = No, 1 = Yes | Defined by ICD-10 code H40 |
| Keratitis | Binary | 0 = No, 1 = Yes | Defined by ICD-10 code H16 |
| Pterygium | Binary | 0 = No, 1 = Yes | Defined by ICD-10 code H11.0 |
| Strabismus | Binary | 0 = No, 1 = Yes | Defined by ICD-10 code H50 |
| Trichiasis | Binary | 0 = No, 1 = Yes | Defined by ICD-10 code H02.0 |
| Uveitis | Binary | 0 = No, 1 = Yes | Defined by ICD-10 code H20/H30 |
| History of ocular surgery | Binary | 0 = No, 1 = Yes | Any prior surgical procedure performed on the eye |
| **Systemic Comorbidities** |  |  |  |
| Diabetes mellitus | Binary | 0 = No, 1 = Yes | Defined by ICD-10 code E10-E14 |
| Hypertension | Binary | 0 = No, 1 = Yes | Defined by ICD-10 code I10 |
| Thyroid disease | Binary | 0 = No, 1 = Yes | Defined by ICD-10 code E00-E07 |
| Sjögren's syndrome | Binary | 0 = No, 1 = Yes | Defined by ICD-10 code M35.0 |
| History of allergy | Binary | 0 = No, 1 = Yes | Self-reported history of systemic allergic reactions or allergic rhinitis |

*Note:* IOP: Intraocular Pressure; ICD-10: International Classification of Diseases, Tenth Revision.

| **Table S2. Comparison of Performance Across Machine Learning Models (Based on 10,000 Samples)** | | | | | |
| --- | --- | --- | --- | --- | --- |
| **Classifier** | **F1 Score** | **AUC** | **Accuracy** | **Sensitivity** | **Specificity** |
| AB | 0.830 | 0.866 | 0.725 | 0.998 | 0.166 |
| LR | 0.885 | 0.862 | 0.803 | 0.980 | 0.181 |
| BAG | 0.899 | 0.928 | 0.826 | 0.993 | 0.229 |
| MLP | 0.900 | 0.937 | 0.828 | 0.997 | 0.238 |
| GBM | 0.906 | 0.934 | 0.837 | 0.996 | 0.246 |
| XGB | 0.943 | 0.966 | 0.898 | 0.995 | 0.343 |
| TabPFN | 0.970 | 0.986 | 0.945 | 0.996 | 0.503 |
| SVM | 0.886 | 0.863 | 0.803 | 0.980 | 0.180 |

*Note:* AB: Adaptive Boosting; LR: Logistic Regression; BAG: Bootstrap Aggregating; MLP: Multilayer Perceptron; GBM: Gradient Boosting Machine; XGB: Extreme Gradient Boosting; TabPFN: Tabular Prior-Data Fitted Network; SVM: Support Vector Machine; AUC: Area Under the Receiver Operating Characteristic Curve.


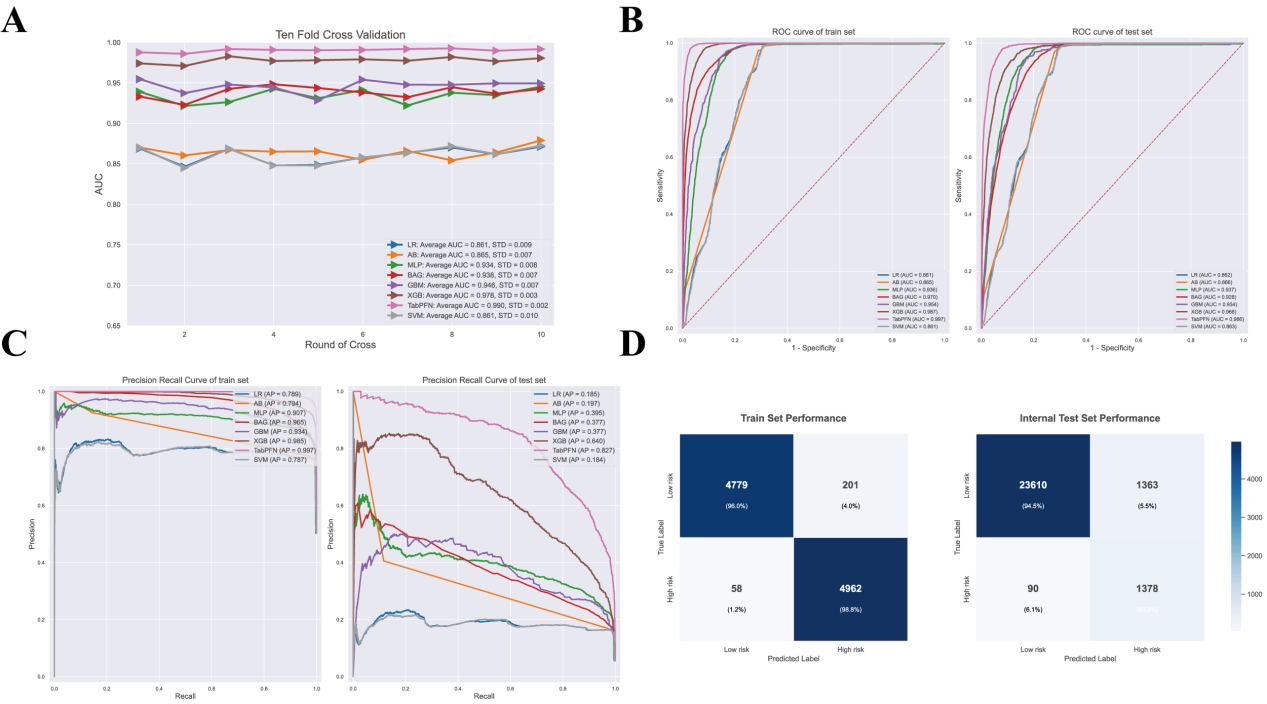


**Figure S1.** Ten-fold cross-validation results on the training set(A), ROC curves (B), and PR curves (C), along with the confusion matrix results (D) for the best ML model.


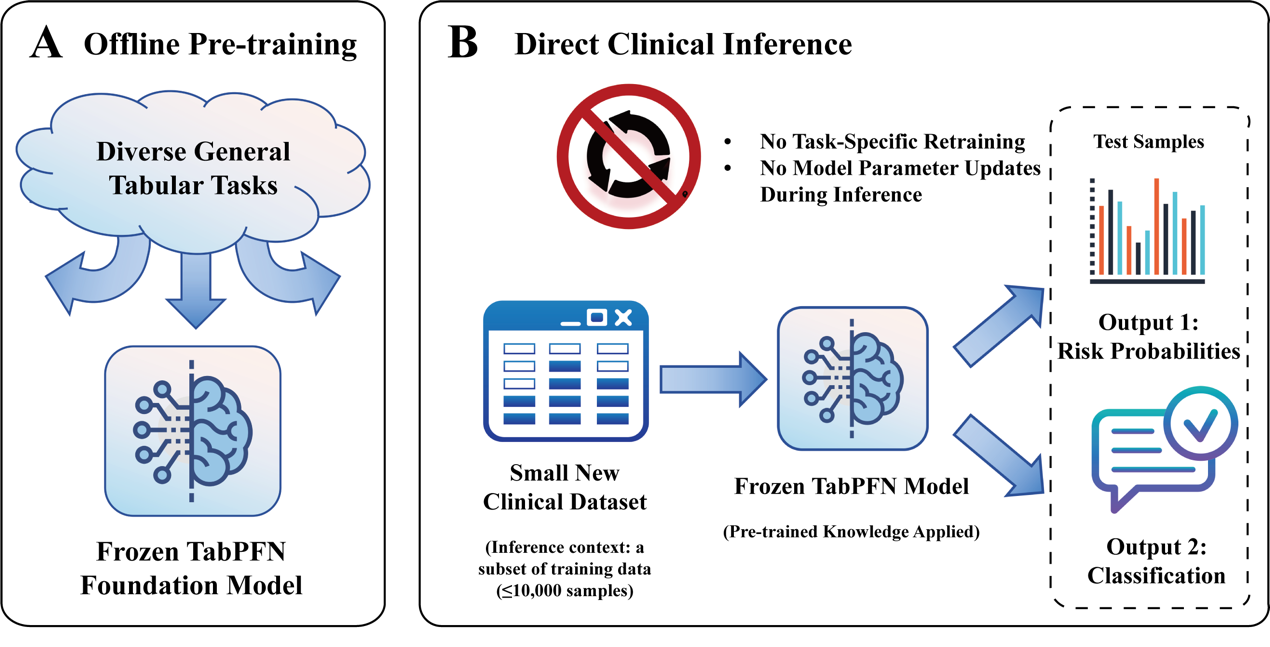


**Figure S2.** The workflow of TabPFN: from offline pre-training to direct clinical inference without task-specific retraining.
